# Efficacy of an Anti-Cellulite Herbal Emgel: A Randomized Clinical Trial

**DOI:** 10.3390/ph14070683

**Published:** 2021-07-16

**Authors:** Ngamrayu Ngamdokmai, Neti Waranuch, Krongkarn Chootip, Katechan Jampachaisri, C. Norman Scholfield, Kornkanok Ingkaninan

**Affiliations:** 1 Centre of Excellence in Cannabis Research, Department of Pharmaceutical Chemistry and Pharmacognosy, Faculty of Pharmaceutical Sciences and Center of Excellence for Innovation in Chemistry, Naresuan University, Phitsanulok 65000, Thailand; ngamrayun59@nu.ac.th; 2 Cosmetics and Natural Products Research Center and Center of Excellence for Innovation in Chemistry, Department of Pharmaceutical Technology, Faculty of Pharmaceutical Sciences, Naresuan University, Phitsanulok 65000, Thailand; netiw@nu.ac.th; 3 Department of Physiology, Faculty of Medical Sciences, Naresuan University, Phitsanulok 65000, Thailand; krongkarnc@nu.ac.th; 4 Department of Mathematics, Faculty of Sciences, Naresuan University, Phitsanulok 65000, Thailand; katechanj@nu.ac.th; 5 Department of Pharmacy Practice, Faculty of Pharmaceutical Sciences, Naresuan University, Phitsanulok 65000, Thailand; norman.scholfield@gmail.com

**Keywords:** anti cellulite, herbal compress, herbal emgel, essential oils, clinical trial

## Abstract

Cellulite describes unsightly skin overlying subcutaneous fat around thighs and buttocks of post-pubescent females. A herbal ‘emgel’ containing volatile oils and extracts of A traditional Thai herbal compress was tested in a double-blind, placebo-controlled trial with 18 women aged 20–50 year with severe cellulite. Appearance of cellulite (primary outcome), thigh circumferences, skin firmness, and cutaneous blood flow (secondary outcomes) were assessed at baseline, 2, 4, 8 and 12 weeks with a 2-week follow-up. Herbal emgel applied onto the thigh skin twice daily reduced cellulite severity scores in every time point. The score was reduced from 13.4 ± 0.3 (baseline) to 12.1 ± 0.3 (week 2) and 9.9 ± 0.6 (week 12). All secondary outcomes improved with both placebo and herbal emgels suggesting that ingredients in the base-formulation might be responsible. Querying of participants, analysis of their diaries, and physical monthly inspections found no adverse events. The herbal emgel safely improved the appearance of cellulite, while the base emgel may play a role for other endpoints. Further studies on the active constituents and their mechanism of action are needed to further explore these factors.

## 1. Introduction

Cellulite describes the lumpy, folded, dimpling, or ‘orange-peel’ appearances of the skin overlying subcutaneous fat, usually around the thighs and buttocks, of post-pubescent females. Gynoidal fat is regarded as an attractive feature in women but when excessive, it is perceived as ugly. It is associated with excessive fat accumulation in these deposits but its etiology is subject to several theories [1]. Most highlight superficial fat, especially gynoidal fat promoted by estradiol via its estrogen receptor alpha (ERa) while fat cells produce estrogen, particularly in obesity. Gynoidal fat is mobilised in pregnancy and lactation but ERa resistance in cellulite may prevent lipid release.

In obesity, the nutritive superficial vasculature and lymphatic vessels become dysfunctional. Excessive fat becomes inflammatory causing fibrosis thereby preventing connective tissue remodeling that accommodates increased dermal and subcutaneous fat deposition which causes the characteristic lumpiness or dimpling [2,3].

Cellulite develops in most women who are not necessarily overweight or obese, and causes no apparent health risk but is aesthetically displeasing. Accordingly, anti-cellulite treatments have proliferated including products based on herbal ingredients. The latter include a wide range of herbal extracts including methylxanthines delivered by subdermal injection, topical creams and wraps, dietary supplements, or alternatively, mechanical interventions such as massaging, laser or light therapy, and liposuction. While they may be efficacious, the invasive approaches carry some risks [4,5]. Adipogenesis or lipogenesis, the differentiation of adipocytes from pre-adipocytes and mesenchymal cells, provide constant renewal of adipocytes and contribute to the increase of adipose tissue mass. As well, insulin plays a predominant role in the adipogenic process. Alternatively, lipolysis and its oxidation safely reduces the adipocyte fat load [6].

The Thai herbal compress is a muslin cloth that encapsulates 100–200 g of mixed herbs and when steamed is used to relieve pain and inflammation. In a previous study, the compress contents were selected to target the main tissue pathologies of cellulite: inflammation, dermal fibrosis, poor microvascular and lymphatic function, and refractory lipolysis [7]. This compress reduced cellulite, skin-fold thickness and circumferences of treated thighs in a placebo controlled trial conducted on 21 premenopausal women for 8 weeks [8]. In vitro, we demonstrated that essential oils and extracts of the herbal compress ingredients reduced lipogenesis and increased lipolysis in 3T3-L1 adipocytes, and were a relaxant in rat aortae [9].

However, herbal compress treatment is labour-intensive and needs dedicated premises. The original compress formulation relied on solid constituents whose active ingredients were leached out by steaming and pressing over the affected skin areas. To offer a more convenient dosage form, the extracts and essential oils comprising mainly monoterpenes from the original formula were emulsified into an aqueous base along with preservatives and a gelling agent to produce a gelled emulsion or ‘emgel’ that adheres to the skin without running off during ~10 min of application.

The objective of the current research was to test the anti-cellulite efficacy and safety of this emgel in a fully blinded, single arm, randomized, placebo controlled trial in women having cellulite.

## 2. Results

### 2.1. Major Constituents of Mixed Essential Oil, Tea and Coffee Extracts

**Figure 1:**
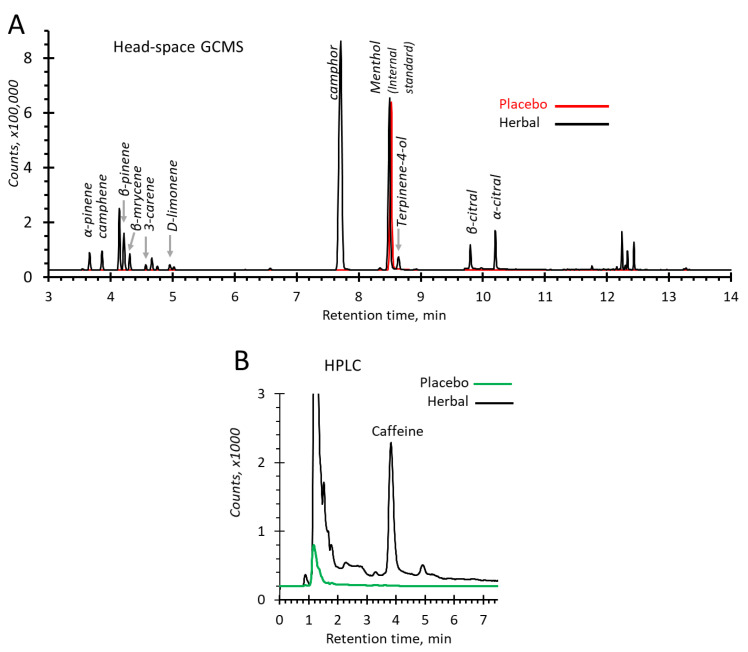
Chromatographic analyses of the chemical constituents in the placebo and the herbal emgels. (**A**) Headspace GC-MS (SIM) total ion chromatograms of 2 mg herbal emgel (black trace), and placebo emgel (red trace, displaced by 2 s), (**B**) HPLC chromatograms of 20 mg/mL of the herbal emgel (black trace), and the placebo emgel (green trace).

Products developed and used in this study were an emulsion and gel mixed to produce an ‘emgel’. The herbal emgel was formulated from the extracts and the mixed essential oils from the herbal compress ingredients considered to be anti-cellulite agents. Ten monoterpenes i.e., camphene, camphor, 3-carene, α-citral, β-citral, limonene, β-myrcene, α-pinene, β-pinene, and terpinene-4-ol, were found as chemical constituents of the herbal emgel analysed by a headspace GC/MS (Figure 1A). These were absent from the placebo emgel that contained no essential oils (red trace, Figure 1A).

Caffeine was also apparent in the herbal emgel (from 0.05% of each tea and coffee aqueous extracts) and identified by retention times using HPLC (Figure 1B).

### 2.2. Participants

**Figure 2:**
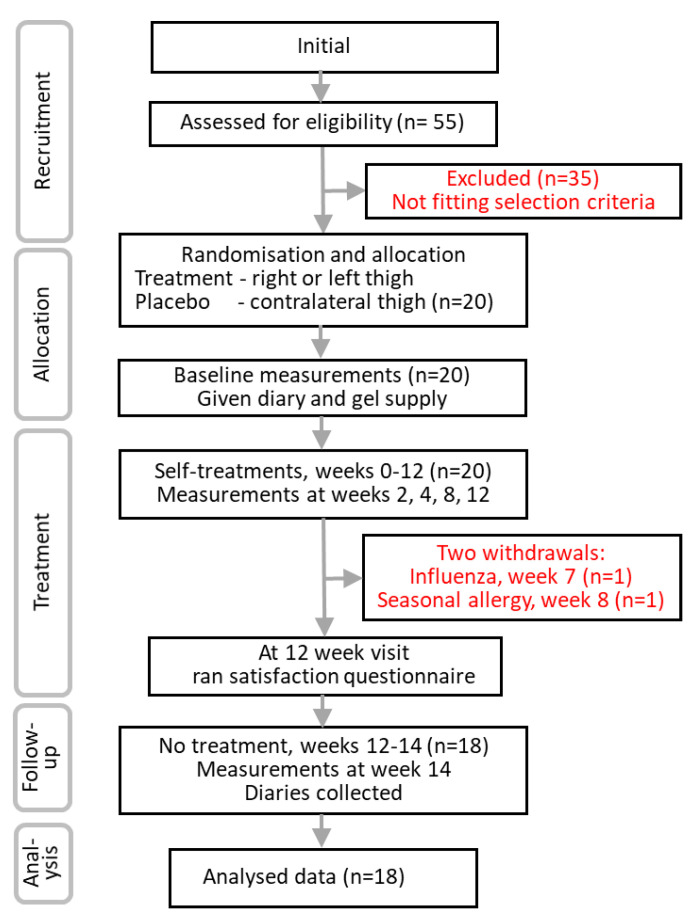
Participant experience through the trial.

Of 55 women answering the advertisement, the 20 who fitted the selection criteria were enrolled, randomized, and instructed in the protocol which they followed (Figure 2). One participant was withdrawn because of an influenza infection (week 7) as was another who developed seasonal allergy (week 8). Both women self-medicated to treat their conditions. Neither were considered to arise from the cellulite treatment.

Eighteen participants completed the trial. None of the participants reported protocol violations and all sachets were returned empty suggesting 100% adherence to the treatment. Their baseline thigh characteristics showed matching of all outcome parameters (Table 1).

### 2.3. Visual Estimation of Cellulite

**Figure 3:**
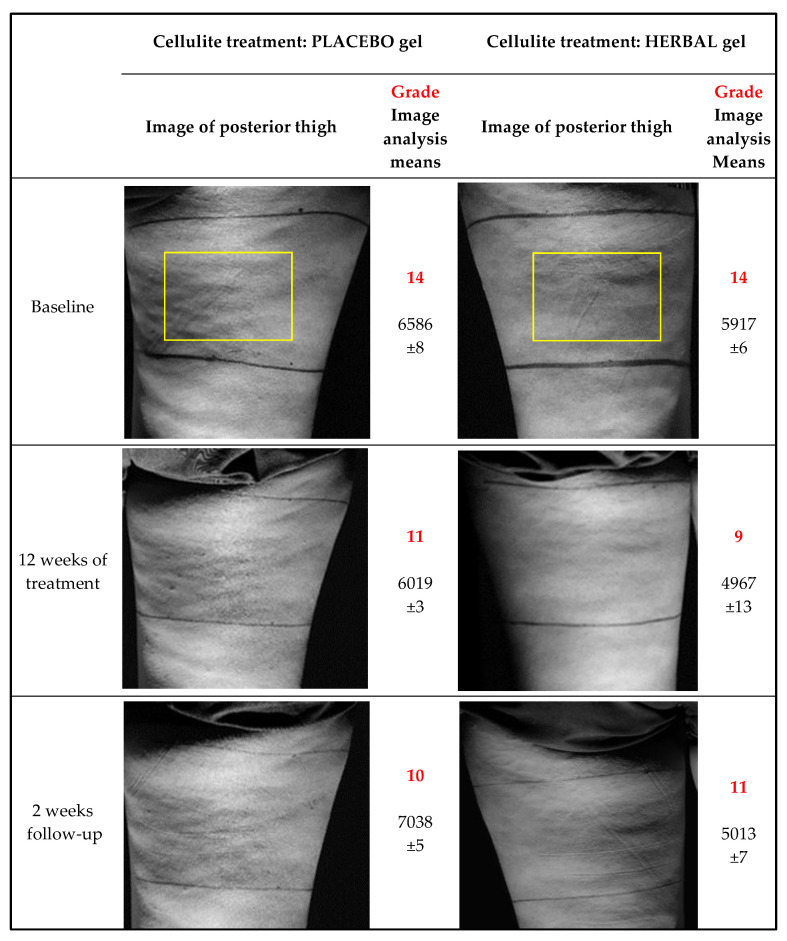
Representative photographs from a single participant showing thighs in posterior aspect as seen by three evaluators whose estimate is given as cellulite grades. The image analysis used the areas bounded by the yellow boxes and the unitless values were generated by three scans of each image to give means ± SEMs.

**Figure 4:**
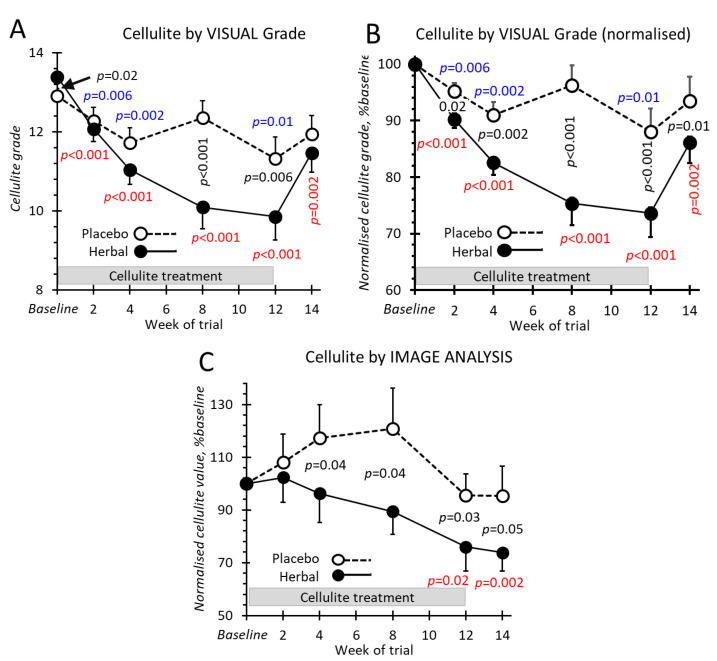
Cellulite changes during the 14 weeks trial. (**A**) Cellulite grades estimated by three blinded independent evaluators. (**B**) Cellulite grades expressed as percentages of the grade before treatment (baseline). (**C**) Normalised cellulite values measured by image analysis. Values are means ± SEMs. *p*-values **in red** compare values for herbal gel with corresponding baseline values, those **in blue** compare values for placebo gel with corresponding baseline values. *p*-values **in black** compare herbal and placebo emgel values at the same time point. All statistical testing was performed on data normalised to baseline values for each participant. An absent value indicates *p* > 0.1. All points are means ± SEM.

Three trained evaluators graded monochrome images similar to examples shown in Figure 3. Treatment with the herbal emgel clearly diminished the observed cellulite over the time-course of the trial (Figure 4) compared to a small reduction produced by the placebo treatment. Cessation of treatment allowed the cellulite to become partly reestablished. The herbal emgel reduced the cellulite grade at week 12 compared to baseline: 1.8 compared to 2.6 found previously [8].

### 2.4. Cellulite by Image Analysis

Reading of the same photographs by image analysis also showed a decline in cellulite reaching about the same level compared to baseline (compare Figure 4B,C). However, the placebo treatment did not show any apparent decrease in the cellulite value using this measurement tool compared with visual grading. These differing details created by the two measuring systems probably arose from the restricted region of interest imposed by the image analysis algorithm (see methods) creating larger variations.

### 2.5. Thigh Circumferences

**Figure 5:**
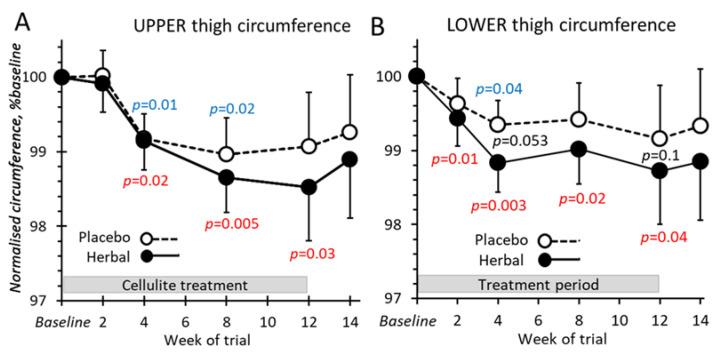
Thigh circumferences 20 (upper) (**A**) or 10 cm (lower) (**B**) above the knee treated with either placebo or herbal emgels. The measurements expressed as the corresponding ratio at time 0 (baseline). For the upper thighs, actual baseline values for placebo and herbal emgels were 55.4 ± 1.1 and 55.6 ± 1.1 cm respectively and 47.1 ± 0.9 and 47.6 ± 1.0 cm for the lower thighs. *p*-value color coded as Figure 4.

Throughout the 12 weeks of treatment, both the lower and upper thighs appeared to become slightly thinner with both placebo and herbal emgel treatments by 0.8–1.0 cm beyond week 2 (Figure 5A,B). There was an apparent additional ~0.5 cm reduction in circumference beyond week 2 up to week 4 with the herbal emgel but this effect failed to reach a significant threshold. Nevertheless, this was probably a genuine action of herbal emgel since: (i) this circumference shrinkage was similar to the previous study [8] and (ii) that all values for herbal emgel were lower at the time points when an effect would be expected (4–14 weeks) and at both 10 and 20 cm above the knees.

### 2.6. Skin Firmness

**Figure 6:**
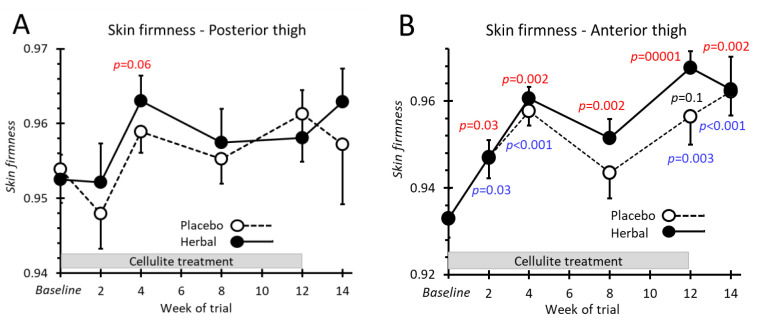
Skin firmness recorded from the posterior (**A**) and anterior (**B**) aspects of the thighs. All *p*-values colour coded as Figure 4.

Skin firmness for the posterior thigh was unaffected by either placebo or herbal emgel (Figure 6A). For the anterior site, both treatments promoted skin firmness but without any superior action by the herbal emgel (Figure 6B).

### 2.7. Cutaneous Blood Flow

**Figure 7:**
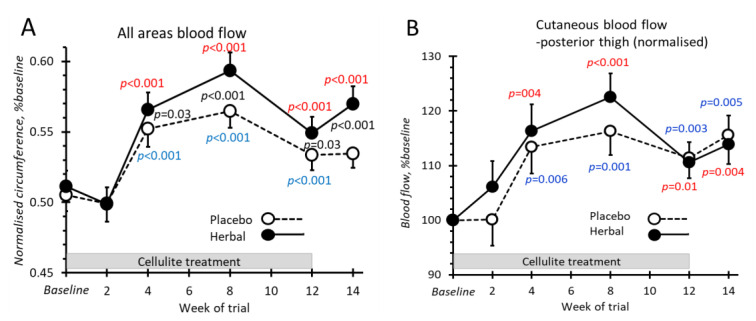
Cutaneous blood flow by laser Doppler flowmetry. All *p*-values colour coded as Figure 3.

Cutaneous blood flow was measured by laser Doppler from eight regions of each thigh (one from each of four sides at 10 cm and another four at 20 cm above the knees). For all the sites tested, cutaneous blood flow increased with both the placebo and herbal emgel treatments after week 2 when compared to the baseline (Figure 7). There was insufficient strength to demonstrate any effect on cutaneous blood flow with herbal emgel.

### 2.8. Participant Comments

Before treatment, participants were questionnaired about their thighs and gave fairly low evaluations that did not discriminate between legs (Table 2).

On completing the trial, 45 spontaneous diary entries were extracted and 16 positive comments were made about the placebo treated thighs compared to 29 about herbal emgel treated thighs, most commonly about firmer skin and reduced cellulite (Table 3).

After the trial, participants were presented with another questionnaire that sought self-assessments about specific properties and actions of the emgels. For the properties, the participants discerned little difference between the two emgels, but expressed some preferences for the herbal emgel actions (Table 4). 

### 2.9. Monitoring and Adverse Events

No adverse events were reported via questionnaires, diaries, or verbal communication. No reddening, swelling, nor irritation were observed around treatment areas by trial personnel at the time of testing.

For clinical monitoring throughout the trial, body weights and BMIs showed no population changes throughout the study but there was a noticeable blood pressure decline (Table 5). Some recruits (n = 10) were postgraduate students who reported stress and sleep deprivation, but the twice daily self-treatment helped relaxation. For this group, BP begin at 114/81 falling to 104/74 at week 12 and for other participants corresponding BPs were 109/74 to 105/72. None suffered from dizziness, faintness or postural hypotension and these values were normal for females of this age group, so was not cause for concern. Blood pressures at follow-up were the same as they were at the end of the treatment period.

## 3. Discussion

The primary endpoint, the appearance of the thigh skin, was improved when treated with the anti-cellulite herbal formulation, as assessed both visually and by image analysis while the placebo showed no effect. The secondary outcomes on thigh circumference, skin firmness, and cutaneous blood flow were considered. For treatment with both placebo and herbal emgels, all three parameters improved while the herbal emgel alone had little further effect over and above the placebo action. A similar placebo effect was observed previously for both cellulite appearance and thigh circumference using herbal compresses [8]. This suggests that herbal emgel is acting on the cellulite appearance alone independent of these other three actions. In explaining these three placebo actions, the following mechanism may operate: (i) one or more of the constituents of the placebo emgel are active, or (ii) a compound in the herbal emgel is transdermally absorbed when applied to one thigh (8% of the body surface) thereby acting systemically on both legs. These are credible possibilities acting alone or in concert but our data is unable to distinguish their contributions. Nevertheless, these arguments support the notion that the improved appearance of cellulite arises from the localised treatment, an assertion supported by our previous study [8] and several other studies [11,12,13,14].

Thigh skin is 2.4 mm thick, while thigh subcutaneous fat is another 12.4 mm [15]. Dermal adipocytes arise from a myofibroblast lineage, are immuno-active in skin wounding, proliferate with cold exposure and dedifferentiate back to myofibroblasts and influence extracellular matrix remodelling [16]. Thus, topically applied anticellulite agents are unlikely to directly influence subcutaneous fat unless systemically absorbed, but could realistically interact with dermal fat.

The mechanisms of the herbal emgel on cellulite have been proposed in the previous studies. The in-vitro adipogenesis inhibition and lipogenesis inhibition of the extracts, the volatile oils, and their monoterpenes ingredients in the emgel have been reported. The mixed volatile oil also demonstrated vasorelaxation activity on rat aortae [9]. In addition, dimethylxanthines in tea and coffee extracts were known to inhibit phosphodiesterase, reduce lipogenesis, and activate lipolysis, helping to restore the normal structure of the subcutaneous tissue and acting as anti-free radicals [17].

We detected an improved cutaneous blood flow, but could not demonstrate a selective effect of the herbal through the topical route. Given this and also given that the herbal is vasodilatory in vitro [9], a dedicated trial on blood flow is needed. For this, nutritive and thermally-activated blood flow need discriminating, blood flow measured in deeper layers (limited to ~0.7 mm with our equipment), and the possible role of systemic absorption and action need to be explored further.

In previous studies, including ours, ~20% cellulite reduction was achieved, suggesting that there is scope for further improvement [18]. While targeting adipocyte lipid turnover is one strategy, only long-term solutions like moderating excessive energy intake or increasing lipid utilisation by oxidative phosphorylation in muscle or mitochondrial uncoupling in beige/brown adipocytes need to be part of, or accompany phytopharmacological interventions.

## 4. Materials and Methods

### 4.1. Formulations

All essential oils of the herbal emgel i.e., ginger (rhizome), black pepper (fruit), java long pepper (fruit), turmeric (rhizome), plai (rhizome), lemongrass (stalk) and kaffir lime (fruit peel) were purchased from Thai-China Flavours and Fragrances Industry Co., Ltd. (Ayutthaya, Thailand). Tea (leaves) (Three Horses^®^) was purchased from Three Horses Tea Co., Ltd. (Bangkok, Thailand). Coffee (seed) was purchased from Coffman International. Co., Ltd. (Bangkok, Thailand). Camphor was bought from TCI Co., Ltd. (Taipei, Taiwan). The formulae of the herbal and placebo emgels are shown in Table 6. The main ingredients of the herbal emgel formulation comprised 5.0% mixed essential oil, 5.0% camphor, 0.05% of tea and coffee extracts. The placebo emgel contained the same base formulation without the essential oil/extracts. These were formulated by the Cosmetics and Natural Products Research Center (Faculty of Pharmaceutical Sciences, Naresuan University).

#### 4.1.1. Tea and Coffee Extracts

Tea or coffee (100 g) of were extracted by boiling in three changes of 400 mL of distilled water. The combined 1200 mL of solution was filtered through a cloth and then centrifuged (100 g, 5 min). The solution was lyophilized to provide dried tea and coffee extracts and stored at −20 °C in screw cap bottles. The ratio of 0.05% tea and 0.05% coffee (aqueous extracts) were present in the emgel treatments.

#### 4.1.2. Mixed Essential Oil

The mixed essential oil contained the essential oils of the herbal ingredients of the herbal compress (ginger, black pepper, long pepper, turmeric, cassumunar ginger, lemon grass and kaffir lime) in the ratio that was reported in the previous study [9].

### 4.2. Quality Control for the Products: Analysis of Monoterenoids by Headspace GC-MS; Caffeine by HPLC

#### 4.2.1. Headspace GC-MS Conditions

Placebo and herbal emgels (2 mg) were weighed in a headspace vial of 20 mL and sealed using a silicone/PTFE septum and then incubated at 140 °C for 5 min. The transfer line temperature was maintained at 250 °C.

Monoterpenoids in the herbal emgel were analysed using a gas chromatograph (Agilent 7890B) and a mass spectrometer (Agilent 5977B MSD) (Agilent Technologies, Santa Clara, CA, USA). The resultant vapour phase injected into the GC/MS in split injection mode (ratio 50:1). The treatment products in the vial had been pressurized with carrier gas, then injected into a capillary column HP-5 5% phenyl methyl silox (30 m × 250 μm × 0.25 μm; Agilent 19091S-433: 93.92873). Helium as the carrier gas flowed at 1.3 mL/min. The initial temperature was held at 75 °C for 1 min and raised at 8 °C/min to reach 100 °C and held for 4 min. The temperature gradient continued at 25 °C/min until 250 °C. The temperature of the injector port was 230 °C and the detector was 250 °C. The total run time was 14.1 min. To identify monoterpenoids, the mass spectrometer was operated in selective ion monitoring (SIM) mode (100 ms dwell times). Electron ionization (EI) system was used with ionization energy at 70 eV. Identities were confirmed by mass spectra retrieved from the National Institute of Standards and Technology (NIST MS Search 2.2 library) spectral database. Menthol (2 mg/mL) was added to the samples as an internal standard prior to the analyses.

#### 4.2.2. HPLC Conditions

To measure caffeine, the placebo and herbal emgels were dissolved in methanol (20 mg/mL) and filtered through nylon syringe filters with a 0.45 μm pore size. The analysis was conducted by using a SCL-10A HPLC system (Shimadzu, Columbia, MD, USA) equipped with a Shimadzu SPD-10A UV/Vis detector, LC-10AT pump, SIL-20AC HT auto-samplers, CTO-10ASVP column oven. For separation, a Phenomenex C18 (10 × 4.6 mm, 5 µm) guard column connected to a Phenomenex Synergi 4μ Hydro-RP 80A column (150 × 4.60 mm, 4 µm particle size) maintained at 35 °C were used (Phenomenex, Torrance, CA, USA). The isocratic mobile phase was methanol and water (40:60 *v*/*v*), flowing at 1.0 mL/min. The injection volume was 10 µL and the eluates were monitored at 275 nm. The total run time was 8 min.

### 4.3. Study Protocol

The protocol was a single arm, double-blind design study where the treatment was randomly allocated to one or other thigh and the placebo applied to the contralateral side and reported according to the CONSORT 2010 guidelines. The participants applied the allocated placebo emgel and then the herbal emgel twice daily (morning and evening) to each thigh for 12 weeks. Morphometric and instrument measurements were carried out before the first application (baseline) and thereafter at 2, 4, 8, and 12 weeks during treatment, and after the 2-week follow-up period without treatment.

### 4.4. Ethical Approval

The protocol was approved by the Institutional Review Board and Ethics Committee, Naresuan University, in accordance with the Helsinki Declaration (2013) and Good Clinical Practices (IRB No. 128/2019, dated 3 April 2019) (COA No.128/2019) and registered with the Thai Clinical Trials Registry, number TCTR20160302001.

### 4.5. Study Site and Setting

The recruitment and clinical study were conducted from May to August 2019. The study was performed in dedicated testing rooms at the Cosmetics and Natural Products Research Center of the Faculty of Pharmaceutical Sciences at Naresuan University, Phitsanulok, Thailand, 65000.

### 4.6. Cohort Size

Twenty participants were needed based on a previous study that generated significance at <0.05. Two sample comparison of proportions power calculated as 80% for total sample size equal to 46 legs (n = 23) suggested a minimum of 18 participants (36 legs).

### 4.7. Outcomes

The primary endpoint was the degree of cellulite scored visually from photographs, and independently by image analysis of photographs. The secondary endpoints were thigh circumferences, cutaneous blood flow, skin firmness, and participant comments.

### 4.8. Participant Specifications

Twenty women who displayed cellulite on the thighs.

### 4.9. Inclusion Criteria

Women aged 20–55 who had cellulite on their thighs. Grade ≥ 2.0 on the Nürnberger and Müller scale, due to subcutaneous fat accumulation, and had a BMI (body mass index) of 20–29, were included.

### 4.10. Exclusion Criteria

Pregnancy, lactation, coagulation disorders, scars, local infections or marks that obscured cellulite on the thighs, systemic diseases, a history of dermatitis and/or allergic reactions to herbs, neuropathy, disorders of the skin or its vasculature, use of hormone contraceptives, anti-histamines, steroids, or had non-steroidal anti-inflammatory drugs within 3 days of beginning the study, anti-cellulite treatment within the past 3 months, or major surgery within the past year [10].

### 4.11. Recruitment

Participants were found by advertisements posted around Naresuan University. Potential participants read the information sheet, had their cellulite assessed, and given a ~15 min briefing. Twenty applicants meeting the selection criteria and signed the informed consent form, were enrolled, and given a diary to log events during the trial.

### 4.12. Criteria for Withdrawal

During the study, participants were withdrawn from the study if they had an adverse reaction or irritation that could result from the intervention, any illness that prevented any period of participation of the study, or used additional treatments for cellulite over the thighs, participated in any other study, became pregnant, or wanted to discontinue the treatment.

### 4.13. Randomization

Each participant was allocated an ID code and treatment allocated to either the left or right thigh by block of four randomization, with the contralateral thigh receiving the placebo treatment.

### 4.14. Blinding and Allocation Concealment

The ID allocation table was securely stored by Dr. Nunguthai Suphrom of the Faculty of Science, who had no role in the treatment formulations, their storage, their distribution to participants, the measurements, nor data analysis. Dr. Nunguthai secured the allocation table and attached appropriate sachet labels that identified the participant ID and to which leg the sachet contents were to be applied. Every measurement was conducted by the same technician throughout the entire trial. All evaluators and technicians were paid, were blinded to allocations, were not authors, and had no other conflicts of interests. All data analyses were deferred until every participant had completed the trial.

### 4.15. Baseline Characteristics

All participants were ethnic Thais and classed by the Asian classification as over-weight (23.0–24.9 kg/m^2^) or obese (25.0–29.9 kg/m^2^), had similar lifestyles, and lived within a 5 km radius of the Cosmetics and Natural Products Research Center at Naresuan University.

### 4.16. Monitoring

Treatment constituents used are common topically applied compounds commonly used in cosmetics. Every day for 1 week and thereafter every 2 weeks for the remainder of the trial each participant was queried about allergies, soreness, or any other health issues arising from the treatment or general health, adherence, variations of daily routines, emgel usability and its odour. Their diaries were examined, and any problems with the trial discussed at every visit. The personal mobile telephone numbers of responsible trial staff were provided for ‘24/7’ contact. Participant body weights and blood pressures were recorded at each visit and the thighs and other skin areas examined for rashes.

### 4.17. Participant Experience

The flow diagram for participant passage through the trial is shown in Figure 2.

### 4.18. Application of the Emgels

Participants applied the emgels twice daily, at 0600–1000 h after a morning shower and at 1800–2200 h after an evening shower, for the 84 consecutive days (12 weeks). Self-treatment began with a shower and thorough drying. They then applied one complete sachet of emgel (5 g) uniformly around the entire circumference of their upper legs from 3 cm above the patella to the inguinal region using gentle circular motions using their palm, until a residual thin oily remained that aided skin penetration (~3 min per thigh). Participants refrained from showering or washing their thighs for at least 30 min thereafter.

### 4.19. Measurement of Clinical Outcomes

Measurements were made at baseline, 2, 4, 8 and 12 weeks, and at a further 2 weeks without treatment (week 14). Participants were asked to wear shorts (‘hot pants’) at visits. During the 14-week study, they were asked to maintain their normal routines and diets, and to avoid anti-cellulite products. Participants were paid expenses of 400 Thai Baht at each attendance (~US$12). Evaluations were conducted in clinical testing laboratories, each dedicated to particular tests under controlled temperature (24 ± 1°C) and humidity (55 ± 2%, limits 65%). Each visit was scheduled at the same time of day. Participants were asked to acclimatise for 30 min in similar conditions and their weights and blood pressures recorded. Then, every participant went through the following series of tests, conducted always by the same technician for each test.

#### 4.19.1. Imaging

The participant stood upright on a rotatable platform with the heel of the leg under test positioned over the axis of rotation and bearing most of the body weight. The other foot was placed ~12 cm in front and 20 cm lateral of the study leg. The thigh aspect under study was illuminated by a white light beam with a 15° dispersion and adjusted to an elevation of ~70° with respect to the participant vertical axis. The light source was positioned at platform level to reduce illumination gradients introduced by a divergent beam. The dedicated camera, an EOS 800D (Canon, Bangkok, Thailand) with auto-exposure, 100 mm focal length, 24 M pixels and 12-bit depth was fixed in position. Each thigh was photographed from posterior, lateral, and anterior aspects and then the contralateral thigh similarly photographed.

Each image was analysed using two methods:

Visual Appearance: Grey scale images were presented separately to three trained judges who graded the severity of cellulite from 0 to 3 according to the Nurnberger and Muller cellulite classification scale [10].

Image Analysis: A region of interest was selected from raw images (4000 × 3000 pixels) that only included one thigh. From this, another region of interest measuring 4 × 60 mm (width × height), referred to the thigh skin, was extracted and vertical line scans converted to 8-bit 2D arrays over the 4 mm width which were then averaged into a single 8-bit vertical array. This array was smoothed using a ‘simple’ algorithm (averaging ~10 pixels shifting 1 pixel at a time along the array) that yielded a pixel array that filtered out features <1 mm in size (Pxarray1). To create an illumination baseline, Pxarray1 was further smoothed by 20 pixels by removing <20 mm features (Pxarray2). Pxarray2 was subtracted from Pxarray1 and summed deviations from the resultant array gave a measure of undulations representing cellulite. Thus, smooth featureless skin yielded a value of 0 and thighs showing visible cellulite had values 200–10000. The software automatically selected 11 more adjacent 4 × 60 pixel regions of interest each time shifted horizontally, and the same algorithm applied each time. Finally, the 12 results were summated into an overall score.

In this simple analysis, cellulite is only apparent as horizontal or diagonal ridges and troughs because the illumination beam is vertical from the bottom. A more serious limitation was that the black lines drawn on the skin, used to measure thigh circumferences, became less distinct and more difficult to digitally oblate over the 14 weeks of the study. This restricted image sampling to a 80 × 60 mm area.

#### 4.19.2. Thigh Circumferences

At the first visit, reference points were marked on the anterior thigh surfaces 10 and 20 cm above the centre of the patella. ‘Velcro’ tape was positioned so that each point around the thigh was 10 or 20 cm vertically above the patella centre. This tape was used as a guide rule to draw a circumferal line on the skin around the thigh with an indelible pen. This line was then used to position a glass fibre flexible tape to measure thigh circumference at ‘10′ and ‘20 cm’ at each visit.

#### 4.19.3. Skin Firmness

This was measured using a Cutometer^®^ MPA 580 (Courage and Khazaka, Köln, Germany) at three repetitions on the posterior thighs [19,20]. The instrument yields a value depending on how much skin is pulled into the probe and varies between 0 (a fluid surface) and 1 (a non-stretchable solid surface). Typical values for skin are close to 1.0 (100%) that provide an indication of cutaneous collagen, elasticity, and shape recovery.

#### 4.19.4. Cutaneous Blood Flow

Blood flow through the cutaneous microcirculation of 6 × 6 cm areas of posterior thighs was imaged by laser Doppler flowmetry using a PeriCam PSI system (Perimed AB, Järfälla, Sweden) using laser speckle contrast analysis. The speckle pattern in the illuminated area was monitored using a 1388 × 1038-pixel CCD camera. Blood perfusion was calculated by analyzing the variations in the speckle pattern in each image pixel. These were averaged for the whole 6 × 6 cm area and provided an estimate of the heterogeneity of cutaneous blood flow [21].

#### 4.19.5. Participant Self-Assessments

A self-assessment questionnaire was collected on day 0 (baseline), which queried the participants view about their skin and cellulite status.

Another questionnaire presented at the week 12 visit sought participant opinions about their subjective perception of formulation efficacies applied to each leg.

At week 14 they completed a further questionnaire seeking their views on the subjective features of the applied formulations, their efficacy, smell, and overall observations about the treatments.

#### 4.19.6. Diary Record

Participants were given a diary containing trial information, scheduled appointment dates, and into which they entered any contemporaneous comments about treatments to each leg, adverse events, and treatment variances.

#### 4.19.7. Data Analyses

The clinical data obtained at each time-point were compared with the baseline for each group and, also between groups, in the search for statistically relevant differences. For continuous outcomes, means ± SD were calculated. Analysis of variance with repeated measures were performed to test the effects of both the placebo and the herbal products over time. Also, thigh circumferences, skin thicknesses, skin firmness, and blood flow microcirculation in each intervention group were compared with the baseline measurements using Bonferroni’s post-hoc test. The satisfaction scores at the trial end were analyzed by Wilcoxon’s test (*p* < 0.05) and the number of participant comments from diary entries recorded were calculated.

## 5. Conclusions

The primary outcome showed a clear amelioration of cellulite of the thigh posterior aspects. Both the herbal and placebo emgels improved the secondary outcomes (thigh circumference, and skin firmness and blood flow) suggesting emgel constituents mediated primary and secondary outcomes. However, the treatment effect sizes are limited without accompanying fat load reductions and increased fat oxidation see with lifestyle changes.

## 6. Patents

The petit patent for the herbal emgel product was obtained from Department of Intellectual Property (DIP), (Thailand), no. 17425, date 11 March 2021.

## Figures and Tables

**Table 1:** Baseline Data.

Parameters	Placebo Emgel	Herbal Emgel
	Means ± SD	
Respondents to advertisements	55	
Participants enrolled	20	
Participants completing the trial	18	
Female	100%	
Age	29.9 ± 8.8 y (22–53)	
Body weight	60.7 ± 12.1 kg	
Body mass index	23.5 ± 3.4 kg/m^2^	
Cellulite grading *	grade 3.0 ± 0	grade 3.0 ± 0
Cellulite Severity Scale (CSS) **	12.9 ± 0.7 (Severe)	13.4 ± 0.8 (Severe)
Cellulite by image analysis ^‡^	8356 ± 3736	8675 ± 3592
Thigh circumference, at 10 cm (Lower)	47.1 ± 4.0 cm	47.6 ± 4.4 cm
at 20 cm (Upper)	55.4 ± 4.6 cm	55.5 ± 4.6 cm
Skin firmness, anterior thigh	0.93 ± 0.02	0.93 ± 0.03
posterior thigh	0.95 ± 0.02	0.95 ± 0.01
Blood flow, posterior thigh	0.40 ± 0.12 PU/mmHg	0.42 ± 0.13 PU/mmHg

* Nürnberger and Müller scale [10]. ** Classification of cellulite based on the results of visual scores (Mild 1–5; Moderate 6–10; Severe 11–15). ^‡^ Image analysis yields a unitless values reflecting the depth of undulations over the skin.

**Table 2:** Pre-trial participant self-evaluation questionnaire about their thighs.

Questions before Treatment about Each Thigh	Self-Evaluations ^a^		
	Left Thigh	Right Thigh	
	Mean ± SD	Mean ± SD	*p*-Value ^b^
The skin feels smooth	2.06 ± 0.80	2.06 ± 0.80	0.500
The skin feels tight	1.56 ± 0.70	1.56 ± 0.70	0.500
The size of my thigh is acceptable	1.72 ± 0.75	1.72 ± 0.75	0.500
The cellulite at my thigh is acceptable	1.78 ± 0.81	1.56 ± 0.70	0.193

^a^ Responses were scored 1 to 5, where 1 = Strongly disagree, 5 = Strongly agree, and 3 = Neutral. ^b^ *P*-value by Wilcoxon signed rank test (2-tailed) comparing left and right thigh.

**Table 3:** Summary of 45 spontaneous free-text comments extracted from participant diaries entered during the 12-week treatment. Translated by sense rather than verbatum.

Generalised Comments about Thighs	Placebo Thigh	Herbal Thigh
	Number of Comments (%)	Number of Comments (%)
My skin seems firmer	4 (22%)	10 (56%)
My cellulite appears to be reduced	5 (28%)	8 (44%)
My thighs appear to be thinner	2 (11%)	4 (22%)
My thigh skin appears to be smoother	5 (28%)	7 (39%)
Total comments	16	29

**Table 4:** Self-evaluation questionnaire about perceptions on cellulite-related properties after 12 weeks of treatment.

The Question Appearing on the Questionnaire—Translated From Thai	Satisfaction Score ^a^		
	Placebo Emgel	Herbal Emgel	*p*-Value ^b^
	Mean + SD	Mean ± SD	
About the emgels			
Gel texture good	3.83 + 0.79	3.50 ± 1.04	−0.14 *
Emgel fragrance pleasant	3.39 ± 1.04	3.11 ± 1.02	−0.21 *
Gel well absorbed by the skin	3.11 ± 1.13	3.06 ± 1.26	−0.44 *
Overall satisfaction with emgel	3.61 ± 0.50	3.56 ± 0.92	−0.41 *
Effects of the emgels			
Your thighs are smoother	3.44 ± 0.98	3.78 ± 0.80	**0.1** **4**
Your thighs feel firmer	3.17 ± 1.04	3.44 ± 1.25	0.24
Your thighs feel smaller	2.83 ± 1.10	3.33 ± 1.08	0.09
Your cellulite appears to be reduced	3.00 ± 1.14	3.61 ± 0.85	0.04
Overall satisfaction with the emgel	3.61 ± 0.85	4.11 ± 0.92	0.04

^a^ The responses were given a numerical value of 1 to 5, where 1 = Strongly disagree, 5 = Strongly agree, and 3 = Neutral. ^b^ *P*-value determined using Wilcoxon signed rank test (2-tailed). * These values in **red** indicate that herbal emgel is worse than placebo emgel, **black** better than.

**Table 5:** Clinical monitoring parameters of participants.

Parameter	Baseline	12 Weeks	*p*-Value
	Means ± SD		
Body weight (kg)	60.4 ± 12.3	60.7 ± 13.3	NS
Body mass index (kg/m^2^)	23.4 ± 3.3	23.5 ± 3.9	NS
Systolic blood pressure, mm Hg	112.0 ± 12.3	104.6 ± 9.5	0.006
Diastolic blood pressure, mm Hg	78.3 ± 9.7	73.4 ± 7.8	0.01

**Table 6:** Composition of the emgels used to treat cellulite.

Ingredient	Herbal Emgel %W/W	Placebo Emgel %W/W
Carbopol	0.8	0.8
Disodium EDTA	0.1	0.1
Propylene glycol	2.0	2.0
Glycerol	6.0	6.0
Microcare (Phenoxyethanol + chlophenesin)	1.0	1.0
Triethanolamine (TEA)	1.0	1.0
Rice bran oil	8.0	8.0
PEG-40 hydrogenated castor oil	5.0	5.0
Tea extract	0.05	0
Coffee extract	0.05	0
Mixed essential oils	5.0	0
Camphor	5.0	0
Demineralized water (aqua)	66.0	76.1

## Data Availability

Data is contained within the article.
